# High Prevalence of Protective HSD17B13 Variant in Taiwan: An Analysis in Morbidly Obese Individuals and Liver Donors

**DOI:** 10.3390/genes17080860

**Published:** 2026-07-24

**Authors:** Hsiao-Yun Lin, Hsiang-Yu Tseng, Chih-Che Lin, Wei-Juo Tzeng, Teng-Yuan Hou, Wei-Feng Li, Yu-Cheng Lin, Shih-Min Yin, Chih-Chi Wang, Yu-Yin Liu, Yu-Hung Lin

**Affiliations:** 1 Division of General Surgery, Department of Surgery, Kaohsiung Chang Gung Memorial Hospital, Chang Gung University College of Medicine, Kaohsiung 833, Taiwan; hylin8629@gmail.com (H.-Y.L.); liuyuyin5750@gmail.com (Y.-Y.L.); 2 Kaohsiung Municipal Feng Shan Hospital-Under the Management of Chang Gung Medical Foundation, Kaohsiung 830, Taiwan; 3 Liver Transplant Center, Kaohsiung Chang Gung Memorial Hospital, Chang Gung University College of Medicine, Kaohsiung 833, Taiwan; 4 Weight Management Center, Kaohsiung Chang Gung Memorial Hospital, Chang Gung University College of Medicine, Kaohsiung 833, Taiwan; 5 Division of General Surgery, Department of Surgery, Kaohsiung Municipal Ta-Tung Hospital, Kaohsiung 801, Taiwan

**Keywords:** Taiwan, genetic, HSD17B13, morbid obesity, non-alcoholic fatty liver disease

## Abstract

Background: We aimed to investigate the prevalence of HSD17B13 polymorphisms while evaluating the correlation between HSD17B13 gene variants and NASH prevalence in the Taiwanese population. Furthermore, we tried to identify the diagnostic value of liver enzymes as non-invasive biomarkers for NASH in morbidly obese patients. Methods: Severely obese patients with non-alcoholic fatty liver disease (NAFLD) who received liver biopsy during bariatric surgery and controls of liver donors who underwent donor hepatectomy were enrolled from 2016 to 2021. Genotyping was utilized in TaqMan PCR assays. For comparison of NAFLD severity, patients were divided into no NASH (NAFLD activity score, NAS 0–2), borderline NASH (NAS 3–4), and NASH (NAS 5–8). Results: The proportion of HSD17B13 rs72613567 TA allele carriers (T/TA and TA/TA genotypes) was 96% in both groups, corresponding to an estimated TA allele frequency of approximately 50% based on a standard allele-counting method. Among morbidly obese patients, T/TA and TA/TA genotypes were associated with lower odds of NASH prevalence compared with T/T genotype. For the non-invasive predictive factors of NASH, NASH group had significantly higher level of liver enzymes compared to “no NASH” and “borderline NASH” groups. Receiver operating characteristic curve analysis revealed there were moderate predictive values for NASH in AST (AUC = 0.71, *p* = 0.014) and ALT (AUC = 0.73, *p* = 0.007). Conclusions: Our study revealed a relatively high prevalence of the loss-of-function TA variant of HSD17B13 in this southern Taiwanese cohort, irrespective of body weight. This variant may contribute to the comparatively lower prevalence of NASH in the region. Additionally, AST and ALT may have potential predictive value for NASH prevalence. Given the single-center design, further larger, population-representative studies are warranted.

## 1. Introduction

In recent decades, non-alcoholic fatty liver disease (NAFLD) has emerged as a significant contributor to the global healthcare burden [1,2]. The global prevalence of NAFLD is estimated at 25–30% in the general adult population, with significant geographic variability [3,4]. Without proper intervention, NAFLD can progress from simple steatosis to non-alcoholic steatohepatitis (NASH), which ranks as the second most common indication for liver transplantation in the USA [5,6]. The gold standard for diagnosing NASH remains liver biopsy. However, due to the invasiveness of liver biopsy and limited immediate treatment options, obtaining histological confirmation of NASH remains challenging in clinical practice. Current clinical practice guidelines increasingly emphasize non-invasive risk stratification to identify patients at high risk of advanced fibrosis [7]. Although several non-invasive biomarkers have been developed and validated—including blood-based tests (FIB-4, ELF, FibroTest) and elastography techniques (VCTE, MRE)—none are routinely used to diagnose NASH in clinical practice [8,9].

Currently, genome-wide association studies (GWAS) have identified relationships between genetic variations and NASH. Genetic polymorphisms—including PNPLA3 I148M, TM6SF2, MBOAT7, and HSD17B13—have been recognized to be associated with more advanced liver disease and HCC development in NASH [3]. 17β-hydroxysteroid dehydrogenase-13 (HSD17B13), one of the lipid droplet-associated proteins in human subjects, is believed to play a significant role in the pathogenesis of fatty liver disease [10]. A protein-truncating variant (rs72613567: TA) in HSD17B13 has been linked to a reduced risk of NASH and fibrosis [11,12,13], with similar protective effects noted in hepatocarcinogenesis and alcoholic liver disease [14,15]. Evidence suggests that the incidence of HSD17B13 varies in different ethnic backgrounds and geographical regions, and this might have an impact on the progression of NAFLD or NASH [11].

In this study, we aimed to investigate the prevalence of HSD17B13 polymorphisms while evaluating the correlation between HSD17B13 gene variants and NASH prevalence in the southern Taiwanese population. Furthermore, we tried to identify the diagnostic value of liver enzymes as non-invasive biomarkers for NASH in morbidly obese patients undergoing bariatric surgery.

## 2. Materials and Methods

### 2.1. Study Design and Eligible Patients

Severely obese patients who underwent bariatric surgery were recruited from our institute from 2016 to 2021. All patients had NAFLD diagnosed by imaging and met the criteria for bariatric surgery according to the Asia-Pacific Bariatric Surgery Group (APBSG) consensus. Patients under 20 years old, those with daily alcohol intake exceeding 42 g/day, use of hepatotoxic drugs, medical history of liver diseases (such as Wilson’s disease, hemochromatosis, α1-antitrypsin deficiency, autoimmune hepatitis, hepatitis B or C, and malignant diseases), and those taking glucose-lowering drugs were excluded from the study. Controls consisted of healthy living liver donors undergoing donor hepatectomy at our institute during the same period.

### 2.2. Clinical and Biochemical Characteristics

Preoperative data, including demographic information, coexisting medical conditions, and anthropometric measurements, were collected using a standardized protocol. Body Mass Index (BMI) was calculated as weight in kilograms divided by the square of height in meters. Severely obese Asian patients with a BMI > 37 kg/m^2^ or a BMI > 32 kg/m^2^ with obesity-related comorbidities were eligible for bariatric surgery. Biochemical data collected including AST, ALT, gamma glutamyl transpeptidase (γ-GT), blood glucose, fasting insulin, total cholesterol, triglycerides, HDL-cholesterol, LDL-cholesterol, and uric acid levels. For the diagnosis of NAFLD, enrolled subjects underwent liver ultrasound or MRI to evaluate fatty liver presence and severity.

### 2.3. Tissue Collection and Histological Assessment

Intraoperative liver biopsy was performed as a standard component in all morbidly obese patients undergoing bariatric surgery during the study period using biopsy needles. Similarly, small wedge biopsies were conducted in living liver donors during donor hepatectomy. These biopsy specimens from both groups were analyzed by experienced histopathologists in the pathology department. Liver samples from the obesity cohorts were routinely stained with hematoxylin and eosin. Steatosis severity was graded on a scale of 0 to 3: (0) steatosis <5%, (1) steatosis 5–33%, (2) steatosis 33–66%, and (3) steatosis >66%. Lobular inflammation was assessed based on the number of inflammatory foci per 200× microscopic field, scored as: (0) no inflammation, (1) 0–2 foci, (2) 2–4 foci, and (3) >4 foci. Ballooning degeneration was graded from 0 to 2: (0) none, (1) few balloon cells, and (2) many balloon cells. The NAFLD activity score (NAS), which is the sum of these scores, classifies NAFLD into non-NASH (NAS 0–2), borderline NASH (NAS 3–4), and NASH (NAS 5–8).

### 2.4. Genotyping

Genomic DNA was extracted from EDTA whole blood samples following standard procedures and stored at −80 °C. For genotyping, 10 ng of DNA was utilized in TaqMan PCR assays (Thermo Fisher Scientific, Waltham, MA, USA) following the manufacturer’s instructions. For the HSD17B13 polymorphisms, A88231392-bp DNA fragment was amplified by polymerase chain reaction for DNA sequencing using the following specific primers:

Forward primer 5′ATGAACATCATCCTAGAAATCCTTC-3′

Reverse primer 5′ATCATGCATACATCTCTGGCTGGAG-3′.

The PCR reactions were initiated with an initial denaturation step at 94 °C for 2 min, followed by 30 cycles consisting of denaturation at 94 °C for 1 min, annealing at 60 °C for 1 min, and extension at 72 °C for 1 min. A final extension was carried out at 72 °C for 10 min. For the analysis of HSD17B13 gene polymorphisms, detection of the HSD17B13 variant was performed using TaqMan SNP Genotyping Assays from Thermo Fisher (Waltham, MA, USA). Allele-specific TaqMan fluorescent probes targeting the HSD17B13 variant, along with a PCR primer pair, were mixed with test samples and analyzed using an ABI 7500 Fast Real-Time PCR System (Applied Biosystems, Foster City, CA, USA).

### 2.5. Statistical Analysis

All statistical analyses were conducted using IBM SPSS Statistics version 20.0 (IBM Corp., Armonk, NY, USA). Graphs were created using GraphPad Prism version 9.0 (GraphPad Software, San Diego, CA, USA). Data are presented with mean ± standard deviation (SD). Differences between groups were assessed for significance using the *t*-test and one-way analysis of variance (ANOVA). The TA allele frequency was estimated from genotype distribution assuming standard allele-counting method. Receiver-operating characteristic (ROC) curves were constructed to estimate cut-off points of biochemical parameters for evaluating NASH prevalence. A two-sided *p* value of less than 0.05 was considered statistically significant.

## 3. Results

### 3.1. Clinical Characteristics of the Subjects

This case–control study included a total of 50 morbidly obese patients who underwent bariatric surgery and 50 living liver donors in the control group. The clinical characteristics are summarized in Table 1. As expected, due to the different surgical indications, obese subjects exhibited a significantly higher preoperative BMI. Additionally, levels of AST, ALT, ALK-P, and γ-GT were elevated in the bariatric surgery group, suggesting a potentially more inflammatory state in obese individuals prone to developing NASH. However, there was no statistical difference observed in total bilirubin levels.

### 3.2. Prevalence of NASH in Morbidly Obese Patients

The severity of fatty liver change and the prevalence of NAFLD were significantly higher (*p* < 0.001) in the obesity group compared to the liver donors (Table 2). Fifty-eight percent of the morbidly obese population exhibited at least borderline NASH, with a median NAFLD activity score (NAS) of 4 and an average liver fat content nearing 40%. No cases of NASH were detected among the liver donors, likely due to the considerably lower degree of fatty liver observed in these highly selective individuals.

### 3.3. Correlation Between BMI and NAFLD Severity

**Figure 1:**
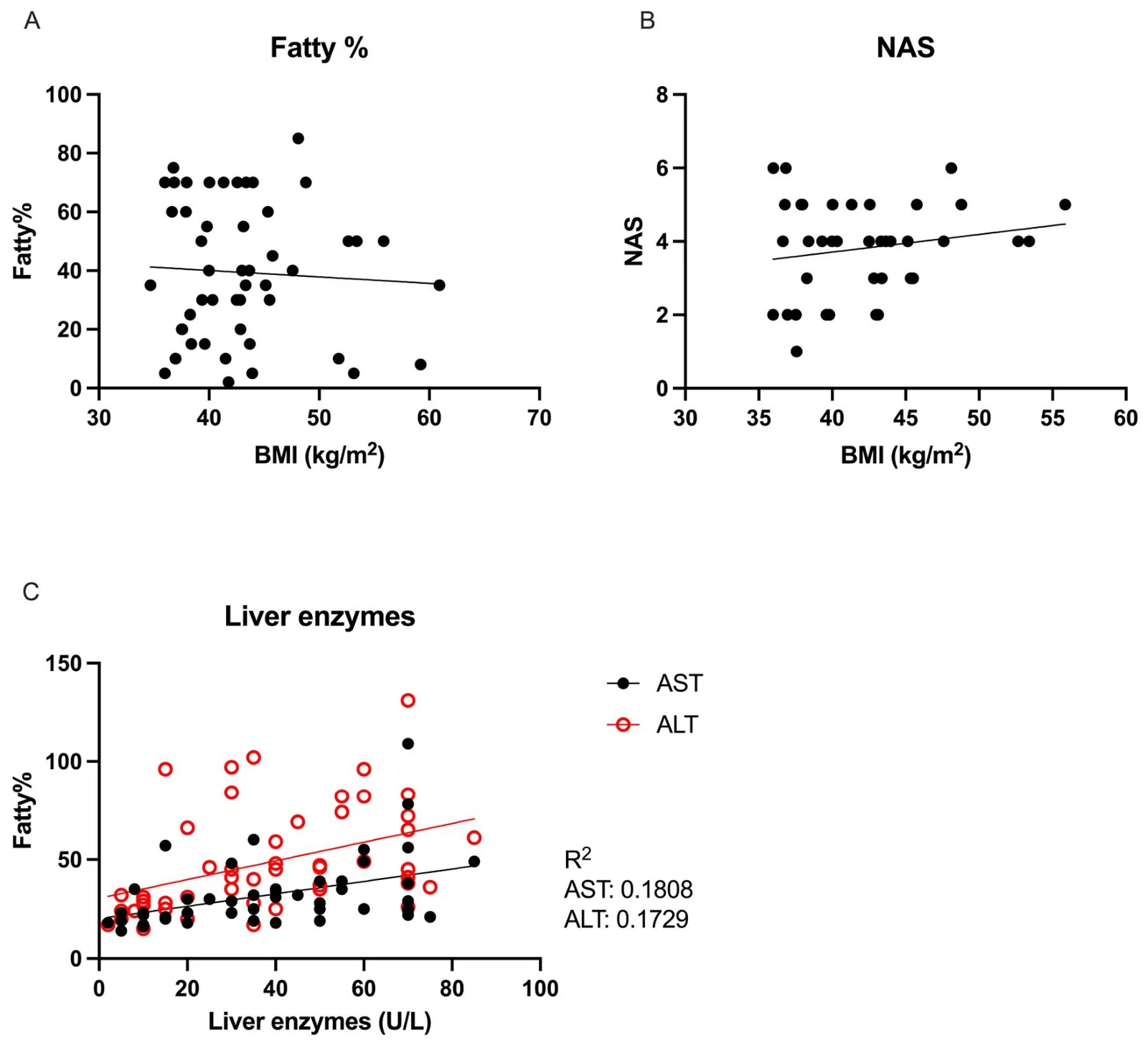
(**A**) Panel A shows the correlation between BMI and fatty (%); (**B**) Panel B shows correlation between BMI and NAS; (**C**) Panel C shows correlation between liver enzymes and fatty (%). BMI, body mass index; NAS, NAFLD activity score.

Obesity has been established as a significant risk factor for NAFLD in recent studies [16]. In the morbidly obese cohort, we found no significant correlation between the degree of liver fat (%) and BMI. While there was a slight positive trend between NAS and BMI, it did not reach statistical significance (Figure 1A,B).

### 3.4. Biochemistry Association with Fatty Liver Histology

In contrast to the histological findings, the results of serum biochemistry showed a slight positive correlation between liver enzymes (AST, ALT) and the degree of liver fat content (Figure 1C). To further investigate the relationship between liver enzymes and other serum biochemistry parameters based on NAFLD severity, we categorized fatty liver disease into three groups according to histological findings: (1) no NASH, (2) borderline NASH, and (3) NASH.

**Figure 2:**
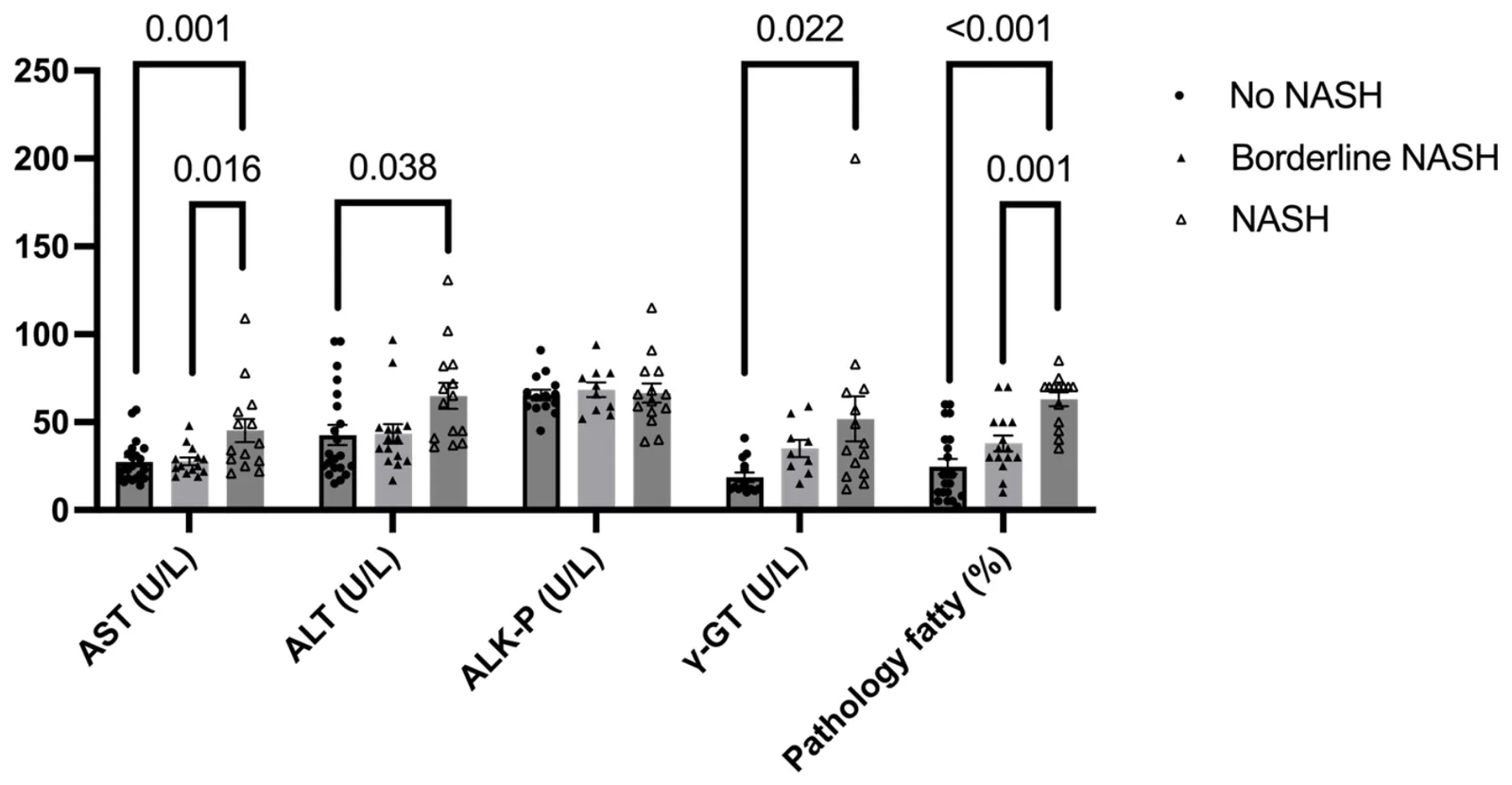
The association between biochemistry and NAFLD severity in morbidly obese patients. AST, aspartate aminotransferase; ALT, alanine aminotransferase; ALK-P, alkaline phosphatase; γ-GT, gamma-glutamyl transferase.

Figure 2 illustrates the percentage of fatty liver change and the levels of serum markers based on histological diagnosis (Figure 2). The results indicated statistically significant higher levels of AST (*p* = 0.005), ALT (*p* = 0.03), and γ-GT (*p* = 0.029) in the NASH group compared to both the “no NASH” and “borderline NASH” groups. Similarly, there was a significant difference in the percentage of fatty liver change (*p* < 0.001).

**Figure 3:**
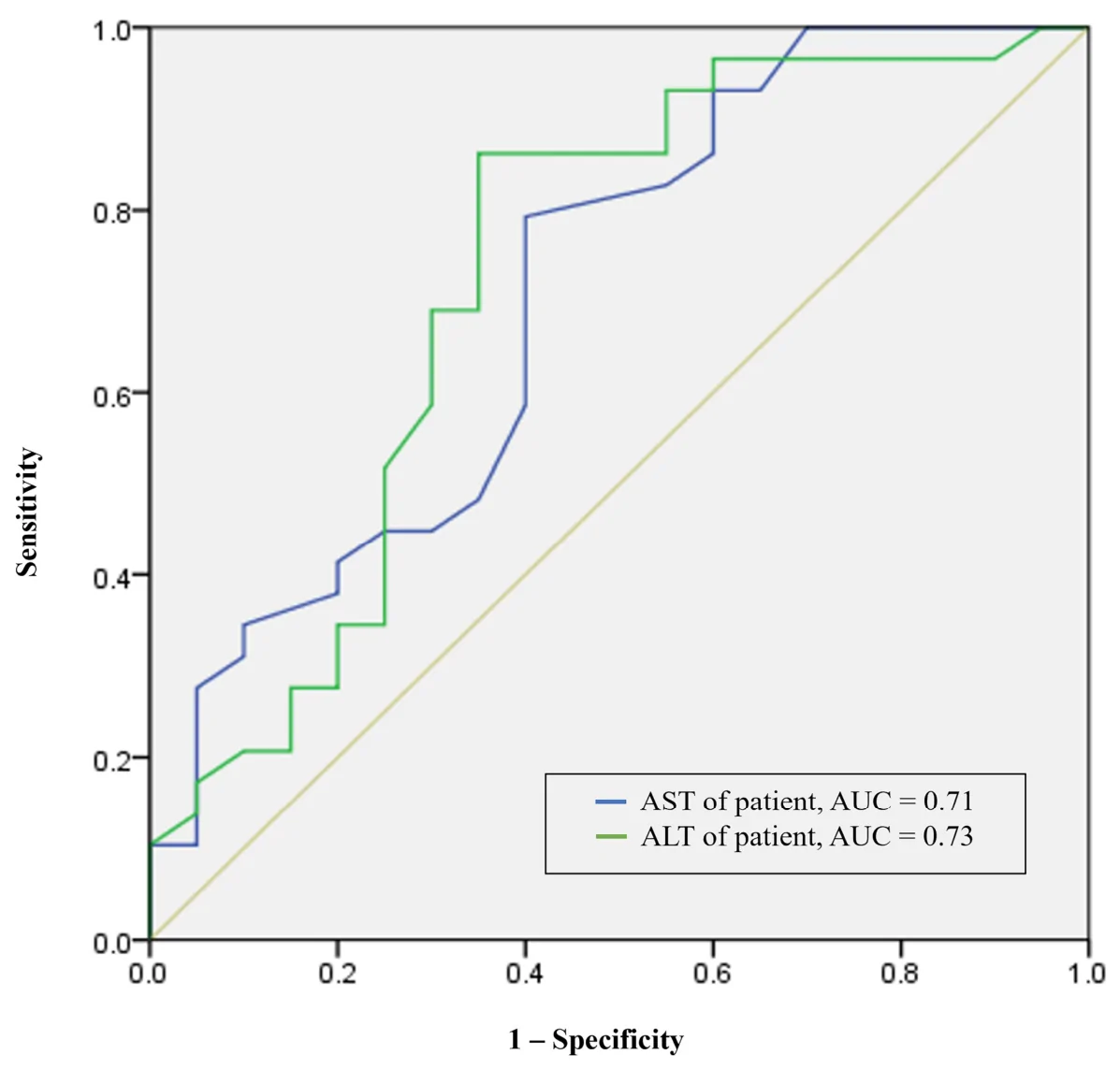
The area under the receiver-operating characteristic curves (AUROC) of AST and ALT for the discrimination of NASH in morbidly obese patient with NAFLD. The diagonal line represents the reference line of no discrimination (AUC = 0.5).

Furthermore, we investigated the diagnostic accuracy of liver enzyme levels for detecting NASH by calculating the area under the receiver operating characteristic (ROC) curve. We observed the area under the receiver-operating characteristic curves (AUROC) of 0.71 (95% CI, 0.56 to 0.86; *p* = 0.014) for AST and 0.73 (95% CI, 0.58 to 0.88; *p* = 0.007) for ALT (Figure 3), indicating moderate diagnostic performance. The cut-off points for AST and ALT were determined to be 23.5 and 33.5, respectively.

### 3.5. Prevalence and Contribution of HSD17B13 Polymorphism

In terms of HSD17B13 polymorphisms, we identified genotype variants in two groups that showed a similar pattern. The T/T genotype carriers accounted for only 4% in both the liver donor and obese subjects groups (Table 3), while the proportion of TA allele carriers (T/TA and TA/TA genotypes combined) was 96% in both groups. Based on standard allele-counting method, this corresponds to an estimated TA allele frequency of approximately 50% in our cohort. Analysis of HSD17B13 polymorphism among morbidly obese patients who underwent bariatric surgery revealed that TT variant carriers had significantly higher liver fat percentage, higher NAFLD activity score (NAS), and higher direct bilirubin levels compared to other genotypes (Table 4). Univariate logistic regression revealed that patients carrying the T/TA or TA/TA genotype had lower odds of NASH compared with T/T genotype carriers (OR = 0.583, 95% CI: 0.46–0.74). This association should be interpreted with caution given the small number of T/T genotype carriers (*n* = 2) in our cohort, which limits statistical power; consistent with this, the chi-square test comparing NASH prevalence across genotype groups did not reach statistical significance (*p* = 0.239).

## 4. Discussion

In recent decades, it has been estimated that one in four individuals globally is affected by NAFLD [17]. The associated morbidity and mortality have contributed to an increasing health burden, particularly among those with morbid obesity. In this study, we identified a predominant prevalence of the protective T/TA and TA/TA genotypes in HSD17B13 polymorphisms, reaching as high as 96% within the general population of southern Taiwan, corresponding to an estimated TA allele frequency of approximately 50%. Additionally, our findings suggested that AST and ALT levels could serve as potential predictors for NASH in a morbidly obese Asian population with NAFLD.

Globally, the prevalence of the TA variant in the HSD17B13 gene is approximately 18%, with the highest frequency observed in East Asians (34%), followed by Europeans (24%), South Asians (16%) and Africans (5%) [6]. Among patients with NAFLD, the TA variant frequency was reported to be 34% in China and 27% in the USA. In our analysis, the estimated TA allele frequency of approximately 50% in southern Taiwan is higher than the previously reported East Asian frequency, and this elevated frequency of the protective TA allele may contribute to the slightly lower prevalence of NASH (58%) observed in our population. Regarding the prevalence of NASH among patients with NAFLD, previous studies estimated global prevalence rates at 59.1%, with regional variations showing Europe at the highest (69.3%), followed by Asia (63.5%) and North America (60.6%) [18,19]. Specifically, within the bariatric surgery population, the global NASH prevalence ranges from 50% to 70% [4]. The reported prevalence in Taiwan (50.6%), which is closely aligned with our observations [20]. This high TA allele frequency was consistent across both the liver donor group and the obesity group. The lack of significant differences in HSD17B13 polymorphism between these two groups may be explained by several factors. Previous studies have shown that the HSD17B13 gene is associated with the progression of chronic liver diseases rather than the initial simple steatosis, which was present in all individuals in our obesity group [11,13]. Notably, a recent study utilizing liver-specific shRNA knockdown of HSD17B13 in obese mice demonstrated that RNAi-mediated HSD17B13 knockdown improved liver health without affecting overall obesity or body weight. This suggests that HSD17B13 primarily influences liver fat metabolism rather than systemic adiposity [21]. Beyond its epidemiological relevance, HSD17B13 rs72613567: TA holds translational value for both risk stratification and therapy. Its dose-dependent protective effect supports potential use in genetic risk scoring, while RNAi-based agents targeting HSD17B13 have shown promising hepatic mRNA knockdown in Phase I trials, reflecting rapid translation toward precision therapy for NAFLD/NASH [22,23,24]. Overall, our findings reveal a relatively high frequency of the protective TA allele in southern Taiwan, regardless of body weight. Further research and extended follow-up are needed to expand upon and clarify these observations.

To better understand potential risk factors of fatty liver disease, we investigated the correlation between BMI, percentage of fatty change, NAFLD activity score (NAS), and liver enzymes. Interestingly, we found no correlation between BMI and either fatty change percentage or NAS. A prospective Australian study involving morbidly obese individuals undergoing bariatric surgery with similar BMI values to our study indicated an independent increase in the odds of NASH with higher BMI categories [25]. Another study highlighted an inverse relationship between BMI and NASH prevalence among subjects with BMI > 50 kg/m^2^, possibly due to adiposity’s protective role in reducing ectopic fat deposition and mitigating liver inflammation [19]. On the contrary, significant associations were observed between elevated liver enzymes and NASH in obese patients compared to those with borderline NASH or non-NASH conditions.

Based on our results, we aimed to determine whether liver enzymes could serve as the non-invasive predictors of NASH prevalence. Despite various parameters proposed for assessing NASH, such as CRP level, splenic longitudinal diameter, Homeostatic Metabolic Assessment (HOMA) index, and cytokeratin 18 (CK18) [17,26], liver biopsy remains the current gold standard, albeit invasive. The diagnostic role of AST and ALT in NASH has been extensively explored in previous studies, yet their definitive diagnostic value remained undetermined. Some studies indicated there was no optimal ALT level for predicting NASH, while others reached different conclusions [27,28]. Supporting our findings, one study established higher cut-off points for both AST (56 IU/L) and ALT (50.5 IU/L) in diagnosing NASH [29]. Nevertheless, the cut-off values in our study (AST = 23.5 IU/L; ALT = 33.5 IU/L) were significantly lower than the traditionally expected threshold for hepatitis. This discrepancy may be explained by revised upper limits suggested in studies for ALT levels in patients with liver disease, setting normal upper limits at 30 IU/L for men and 19 IU/L for women [30,31]. This suggests that clinicians should maintain a high suspicion for NASH in morbidly obese patients, even when liver enzymes appear normal.

One of the strengths of our study was that histological diagnosis via liver biopsy provided reliable results in determining NASH prevalence. However, our study had several limitations. Firstly, it was conducted at a single center with a relatively small sample size (*n* = 100 in total), which limits the statistical power, particularly given the low incidence of the T/T genotype in both study groups. Secondly, our control group consisted of healthy living liver donors, which may not represent a random sample of the general population.

## 5. Conclusions

Our study identified a relatively high prevalence of the loss-of-function TA variant of HSD17B13 in this southern Taiwan population, irrespective of body weight. Furthermore, serum AST and ALT levels demonstrated moderate discriminatory performance for NASH and may serve as potentially useful, non-invasive screening indicators to raise clinical suspicion in the morbidly obese population. To further elucidate the role of HSD17B13 in fatty liver-related outcomes, larger, population-representative studies with long-term follow-up are strongly recommended.

## Figures and Tables

**Table 1:** Baseline clinical characteristics.

Characteristics	Liver Donor (*n* = 50)	Obesity (*n* = 50)	*p* Value
Age	35.1 ± 9.8	37.1 ± 10.0	0.32
BMI (kg/m^2^)	25.3 ± 3.6	43.1 ± 6.1	<0.001
AST (IU/L)	18.3 ± 5.9	32.6 ± 17.5	<0.001
ALT (IU/L)	19.2 ± 12.0	49.1 ± 26.7	<0.001
ALK-P (IU/L)	59.2 ± 15.2	66.6 ± 15.2	0.029
Total bilirubin (mg/dL)	0.6 ± 0.3	0.7 ± 0.3	0.068
Creatinine (mg/dL)	0.7 ± 0.2	0.9 ± 0.3	0.026
γ-GT (IU/L)	13.2 ± 12.2	35.1 ± 33.6	<0.001

BMI, body mass index; AST, aspartate aminotransferase; ALT, alanine aminotransferase; ALK-P, alkaline phosphatase; γ-GT, gamma-glutamyl transferase; Normal reference ranges at our institution’s central laboratory: ALT, <41 IU/L; AST, <37 IU/L; ALK-P, 40–130 IU/L; Total bilirubin, 0.2–1.3 mg/dL; Creatinine, 0.7–1.3 mg/dL for males and 0.5–1.1 mg/dL for females; γ-GT, <60 IU/L for males and <35 IU/L for females.

**Table 2:** Fatty change% and NAFLD activity score (NAS).

	Liver Donor (*n* = 50)	Obesity (*n* = 50)	*p* Value
Number	50	50	
Fatty change (%) (mean ± SD)	2.4 ± 3.1	39.4 ± 23.5	<0.001
NAS (median [range])		4 [1–6]	
No NASH		21 (42%)	
Borderline NASH		14 (28%)	
NASH		15 (30%)	

NAFLD, Non-alcoholic fatty liver disease; NAS, NAFLD activity score; NASH, non-alcoholic steatohepatitis.

**Table 3:** HSD17B13 polymorphism prevalence.

Genotype	Liver Donor (*n* = 50)	Obesity (*n* = 50)
T/T	2 (4%)	2 (4%)
TA/TA	2 (4%)	1 (2%)
T/TA	46 (92%)	47 (94%)

**Table 4:** HSD17B13 polymorphism comparisons.

Variants	TA/TA, T/TA (*n* = 48)	T/T (*n* = 2)	*p* Value
Fatty%	38.1 ± 23.1	70 ± 0	<0.01
NAS	3.8 ± 1.3	5 ± 0	<0.01
BMI	43.1 ± 6.1	43.4 ± 7.7	0.942
AST	31.7 ± 16.5	53.5 ± 34.6	0.084
ALT	48.7 ± 27	58.5 ± 19.1	0.614
ALK-P	65.5 ± 14.9	85 ± 8.5	0.077
Bilirubin, Total	0.7 ± 0.3	0.6 ± 0.1	0.471
Bilirubin, Direct	0.169 ± 0.1	0.20 ± 0	0.006
Creatinine	0.9 ± 0.3	0.6 ± 0	0.212
γ-GT	34.8 ± 33.8	40.5 ± 40.3	0.819

NAS, NAFLD activity score; BMI, body mass index; AST, aspartate aminotransferase; ALT, alanine aminotransferase; ALK-P, alkaline phosphatase; γ-GT, gamma-glutamyl transferase.

## Data Availability

The data described in the manuscript are available from the corresponding authors upon reasonable request.

## Abbreviations

The following abbreviations are used in this manuscript:

ALT	Alanine transaminase
AST	Aspartate transferase
BMI	Body mass index
CK18	Cytokeratin 18
NAFLD	Non-alcoholic fatty liver disease
NAS	NAFLD activity score
NASH	Non-alcoholic steatohepatitis
γ-GT	Gamma glutamyl transpeptidase
17β-HSD13	17β-hydroxysteroid dehydrogenase-13
